# Intrinsic capacity rather than intervention exposure influences reversal to robustness among prefrail community-dwelling older adults: A non-randomized controlled study of a multidomain exercise and nutrition intervention

**DOI:** 10.3389/fmed.2022.971497

**Published:** 2022-10-21

**Authors:** Laura Tay, Ee-Ling Tay, Shi Min Mah, Aisyah Latib, Yee-Sien Ng

**Affiliations:** ^1^Geriatric Medicine, Department of General Medicine, Sengkang General Hospital, Singapore, Singapore; ^2^Geriatric Education and Research Institute, Singapore, Singapore; ^3^Department of Physiotherapy, Sengkang General Hospital, Singapore, Singapore; ^4^Centre for Population Health Research and Implementation, SingHealth, Singapore, Singapore; ^5^Department of Rehabilitation Medicine, Singapore General Hospital, Singapore, Singapore

**Keywords:** intrinsic capacity, frailty, prefrailty, exercise, nutrition, elderly

## Abstract

**Background:**

The differential risk profiles associated with prefrailty may be attributable to underlying intrinsic capacity (IC).

**Objectives:**

We examine (i) effect of a multi-domain physical exercise and nutrition intervention on pre-frailty reversal in community-dwelling older adults at 1-year, and (ii) whether IC contributes to pre-frailty reversal.

**Methods:**

Prefrail participants in this non-randomized study were invited to attend a 4-month exercise and nutritional intervention following a frailty screen in the community. Prefrailty was operationalized as (i) FRAIL score 1–2 or (ii) 0 positive response on FRAIL but with weak grip strength or slow gait speed based on the Asian Working Group for Sarcopenia cut-offs. Participants who fulfilled operational criteria for prefrailty but declined enrolment in the intervention programme served as the control group. All participants completed baseline IC assessment: locomotion (Short Physical Performance Battery, 6-minute walk test), vitality (nutritional status, muscle mass), sensory (self-reported hearing and vision), cognition (self-reported memory, age- and education adjusted cognitive performance), psychological (Geriatric Depression Scale-15, self-reported anxiety/ depression). Reversal of prefrailty was defined as achieving a FRAIL score of 0, with unimpaired grip strength and gait speed at 1-year follow-up.

**Results:**

Of 81 participants (70.0 ± 6.6 years, 79.0% female), 52 participants (64.2%) were enrolled in the multi-domain intervention, and 29 participants (35.8%) who declined intervention constituted the control group. There was no difference in age, gender and baseline composite IC between groups. Reversal to robustness at 1-year was similar between intervention and control groups (30.8% vs. 44.8% respectively, *p* = 0.206). Reduced prevalence of depression was observed among participants in the intervention group at 1-year relative to baseline (7.8% vs. 23.1%, *p* = 0.022). In multiple logistic regression, intervention had no effect on prefrailty reversal, while higher composite IC exhibited reduced likelihood of remaining prefrail at 1-year (OR = 0.67, 95% CI 0.45–1.00, *p* = 0.049).

**Conclusion:**

Focusing only on the locomotion and vitality domains through a combined exercise and nutritional intervention may not adequately address component domain losses to optimize prefrailty reversal. Future studies should examine whether an IC-guided approach to target identified domain declines may be more effective in preventing frailty progression.

## Introduction

Healthy aging has been presented by the World Health Organization (WHO) as the process of developing and maintaining the functional ability to enable well-being in older age (1). Intrinsic capacity (IC), represented by the composite of physical and mental reserves, is central to functional ability through its interaction with the environment (2). Underpinned by the International Classification of Functioning, Disability and Health framework, the construct of IC is supported by five health-related domains: locomotion, vitality, sensory function, cognition and psychological function (3). Pathways addressing these five domains have been developed in the Integrated Care for Older People as an evidence-based strategy for optimizing IC and maintaining functional ability (4).

Since its inception, cumulative evidence has supported declining IC as being representative of the diminished homeostatic reserves underpinning the excess vulnerability typical of frailty (5–7). Accordingly, IC and frailty should thus be considered complementary in their common goal of advancing disability prevention through the maintenance of functionality (8). It is also notable that both frailty and IC are multi-dimensional and dynamic. Domains of IC have been included as key characteristics in both phenotypic and cumulative deficit models of frailty (9, 10). Interventions seeking to reverse frailty or improve health outcomes for frail older adults have largely focused on exercise, nutritional intervention or multicomponent strategies such as the combination of exercise, nutrition and cognition training (11–13). In parallel, a recent review of multidomain lifestyle interventions suggested heterogeneous outcomes for the different IC domains, with benefits on locomotion and, to a lesser extent, cognition and vitality (14).

Prefrailty has been recognized as a prodromal state along the frailty continuum. While the consensus established that prefrailty is reversible, the process is likely non-linear with dynamic transitions and trajectories between robust, prefrail and frail states (15). Further, prefrailty may not be a homogenous biological syndrome, evident by varying outcomes among people identified as prefrail (16). The variable risk profiles of prefrail older adults for frailty development may be attributable to underlying IC, as suggested by a recent cluster analysis in which prefrail older adults could be associated with either intermediate or low IC, with the latter exhibiting greatest risk for decline in physical and functional performance at 1-year (17). This stratification of prefrail older adults by IC with differential risk profiles further underscores the complex health needs of prefrailty. However, the extent by which IC may impact on the outcomes of prefrail older adults exposed to interventions designed to prevent frailty progression has yet been evaluated.

We aim to examine (i) effect of a multi-domain physical exercise and nutrition intervention on pre-frailty reversal in community-dwelling older adults at 1-year, and (ii) whether IC contributes to pre-frailty reversal.

## Methods

### Study design and participants

For this non-randomized controlled trial of a multi-domain physical exercise and nutritional intervention, potential prefrail participants were identified through our ongoing community frailty screening “Individual Physical Proficiency Test for Seniors (IPPT-S),” which had been previously described (18). In brief, the mobile screening platform is based at the void decks of public housing blocks, senior activity centers, and community clubs in the Northeastern region of Singapore served by a regional healthcare facility, and participants in the programme return for yearly follow-ups. Any individual who is aged ≥55 years, community-dwelling and able to ambulate independently (with or without walking aid) is eligible. Residents of sheltered or nursing homes, and persons who are unable to ambulate for at least four meters independently are excluded. Participants meeting operational criteria for prefrailty were invited to attend the 4-month multidomain intervention programme. Ethics approval was obtained from Singhealth Institutional Review Board and all participants provided written informed consent. The study is registered with ClinicalTrials.gov (Identifier: NCT04656938).

### Clinical assessment

All participants completed a multi-domain geriatric screen and physical fitness assessment administered by trained study team members at baseline and 1-year follow-up. The multi-domain geriatric screen included risk factors for frailty—mood (Geriatric Depression Scale-15, GDS-15), cognition (locally validated version of the Chinese Mini Mental State Examination, CMMSE), and nutrition (Mini Nutritional Assessment- Short Form, MNA-SF) (19–21). Functional performance was assessed using Barthel Index (BI) for activities of daily living (ADLs) and Lawton and Brody's scale for instrumental ADLs (22, 23). The University of Alabama Life Space Assessment (LSA) was adopted as a measure of functional mobility (24), while Physical Activity Vital Sign (PAVS) was used to quantify engagement in moderate to vigorous-intensity physical activity (walking, cycling, jogging, swimming, Tai Chi, golf, and housework), documenting the time spent on each activity in the preceding 7 days (25). We assessed social vulnerability based on socio-economic status (self-reported adequacy of expenses) and social support (living alone, availability of a confidant and maintaining social contact with friends or relatives). Medical comorbidities were recorded based on self-reported physician diagnoses of hypertension, diabetes mellitus, malignancy, chronic lung disease, heart disease (myocardial infarction, angina), congestive heart failure, chronic kidney disease, stroke, asthma, and arthritis.

The physical fitness test battery was modified from the Senior Fitness Test (26), and participants who reported feeling unwell on pre-assessment screening were exempted. Measures of physical fitness included (i) gait speed (10 m-walk at usual pace), (ii) grip strength (JAMAR hand dynamometer, with 2 trials for each hand and maximal value used for analysis), (iii) upper and lower limb flexibility (Back Scratch and Chair Sit-and-Reach tests) (27), (iv) upper limb dexterity (Box-and-Block test) (28), (iv) lower limb strength and power (30-second chair stand test) (29), (v) balance (Timed-Up-and-Go test) (30) and (vi) cardiorespiratory endurance (6-minute walk test) (31). Additionally, all participants were scored on the Short Physical Performance Battery (SPPB) (32).

Body composition was measured using multi-frequency segmental Bioelectrical Impedance Analysis (BIA, MC-780 M, TANITA, Tokyo, Japan), with appendicular skeletal mass index (SMI) calculated as the sum of fat-free lean mass of all 4 limbs divided by height-squared (ASM/ht^2^). Low muscle mass was defined using Asian Working Group for Sarcopenia 2019 (AWGS2019) cut-off values of <7.0 kg/m^2^ for men and <5.7 kg/m^2^ for women (33).

### Deriving an intrinsic capacity score

Measures representative of the 5 domains of IC—locomotion, vitality, sensory, cognitive, and psychological—were derived from the multi-domain geriatric screen and physical fitness assessment (7). Each domain was scored on a 3-point scale (0–2), and all 5 domains summated to yield a composite IC score (range 0–10). Locomotion was based on the Short Physical Performance Battery (SPPB, range 0–12) consisting of chair-stand, gait speed and standing balance, and the 6-minute walk test (6 MWT) (31, 32). A score of ≤9 on SPPB, and total distance walked of <400 m in 6MWT were considered impaired performance for the respective tests. Locomotion domain was scored as 0 (impaired performance in both SPPB and 6 MWT), 1 (impaired performance in either SPPB or 6MWT) or 2 (both SPPB and 6 MWT unimpaired). Vitality was represented by nutritional status and appendicular skeletal muscle mass (ASM). In the Mini Nutritional Assessment-Short Form questionnaire (MNA-SF, range 0–14), a score of 8–11 indicates being at-risk of malnutrition, while <8 indicates being malnourished (21). Low muscle mass was defined using AWGS2019 cut-off values (33). The vitality domain was scored as 0 to 2, with a score of 0 assigned for participants who were both at-risk of malnutrition/ malnourished and had low muscle mass, 1 when either at-risk of malnutrition/malnourished or demonstrating low muscle mass, and 2 with normal nutritional status and normal muscle mass. Sensory domain was assessed using self-reported responses to the questions “problems due to poor hearing” and “problems due to poor vision.” Participants with a positive response to both hearing and visual problems scored 0, those reporting either hearing or visual problems scored 1, while those with neither hearing nor visual problems scored 2 in the sensory domain. Cognitive domain was evaluated using both subjective report and performance on the modified Chinese version of the Mini Mental State Examination (CMMSE, range 0–28). Participants responded with yes or no to the question “Do you feel you have more problems with memory than most?”. We used locally validated age- and education-thresholds to define impaired cognitive performance on the CMMSE (21 and 24 for participants <75 years with 0–6 and ≥6 years of education; 19 and 23 for participants ≥75 years with 0–6 and ≥6 years of education) (20). The cognition domain was scored as 0 for participants with CMMSE performance below threshold values for their age and education, 1 for participants with subjective memory problems but unimpaired CMMSE performance, and 0 for participants reporting no memory problem and unimpaired CMMSE. Psychological domain was assessed using the 15-item Geriatric Depression Scale (GDS-15, range 0–15), and a single question from the EuroQol-5 Dimensions (EQ-5D) question on anxiety/depression. GDS-15 score ≥5 suggests depression (19), while the EQ-5D question was assigned scores from 0 (not anxious/depressed) to 4 (extremely anxious/depressed) (34). The psychological domain was scored as 0 for participants with GDS-15 ≥5, 1 when EQ-5D anxiety/depression ≥1 but GDS-15 <5, and 2 for participants with GDS-15 <5 and EQ-5D anxiety/depression = 0.

### Operationalizing prefrailty for intervention

All participants completed the FRAIL scale (35), with 1 point assigned for each positive response—Fatigue, Resistance, Ambulation, Illnesses, and Loss of weight. The identification of prefrailty for intervention was based on (i) 1 or 2 positive responses on the FRAIL questionnaire or (ii) 0 positive response on FRAIL but with weak grip strength (<26 kg for males; <18 kg for females) or slow gait speed (<0.8 m/s) based on the Asian Working Group for Sarcopenia (36).

Participants were considered as having reversed to non-frail state at 12-month if FRAIL score was zero AND grip strength as well as gait speed were unimpaired based on AWGS cut-offs (36).

### Intervention

Eligible pre-frail participants were invited to enroll in a 4-month multi-disciplinary intervention programme comprising (i) once-weekly group-based exercise classes lasting 1 h each session (total of 16 sessions) with individually prescribed home exercises for maintenance between sessions and (ii) group-based nutritional education (6 sessions). Group size was maintained at 8–10 participants to ensure that each participant received adequate attention. While the intensity of exercise was not measured, the target was to achieve at least moderate intensity as tolerated by the seniors. The exercises focused on strength, balance and endurance training, with a warm-up and cool-down routine. TheraBands and step boards were used for resistance and balance training, respectively. The exercise sessions were designed for progressive intensity (such as increasing number of repetitions, increased resistance of the TheraBands, height of step boards) based on the group's progress, while catering for individual variability, with group sessions conducted under the supervision of a physiotherapist or an exercise physiologist. Each session commenced with 5 min of dynamic warm-up e.g., slow marching with small to big arm circles, followed by 45 min of exercises focusing on: (a) balance, coordination and speed e.g., heel to toe walks; (b) strength e.g., rising from a chair and Theraband exercises for lower and upper limbs, respectively; and (c) endurance e.g., fast walking. All sessions ended with 5 min of static cool down e.g., stretching muscles of the thigh and arms with slow breathing. Individually prescribed structured home exercise folders comprising pictorials and written explanations were provided at the end of each session, to encourage participants to maintain regular physical exercise between the group classes. Compliance with home exercise was tracked using a weekly diary, and participants were instructed to achieve a target of performing the prescribed home exercises at least 3 days each week. The nutritional intervention was delivered with the aim of facilitating healthy eating habits to achieve adequate protein, energy, calcium and Vitamin D through regular food and beverages that are more specific to the Asian palate. There were 6 sessions over the 4-month intervention period with 2 sessions per month in the first and second months and 1 session per month in the third and fourth months. Each session lasted 1.5 h and was delivered by a trained nutritionist, incorporating a combined modality of teaching methods that included didactics, food-based games and grocery-shopping trips with sponsored vouchers to provide guidance on choosing quality foods within budget.

### Control

Participants who fulfilled operational criteria for prefrailty but declined enrolment in the intervention programme following IPPT-S screening served as the controls. As part of the IPPT-S screening, these participants (as well as those in intervention group) would have received an individual counseling session with a member of the study team, providing feedback on the results of their screening, along with advice on physical activity and nutrition for frailty prevention reinforced in a personal frailty booklet. They were also invited to attend 4 group-based education classes on frailty that were conducted within the senior activity centers after each screening cycle.

### Outcomes

Reversal of prefrailty was defined as achieving a FRAIL score of 0, with unimpaired grip strength and gait speed at 1-year follow-up. Hospitalization and falls during the 1-year period were captured based on self-report.

### Statistical analysis

Descriptive data are presented as means (±SD) or median (interquartile range, IQR) for quantitative variables and as absolute and relative frequencies for categorical variables. Chi-squared and *t*-tests or Wilcoxon rank-rum tests were used for univariate analyses comparing intervention and control groups in baseline characteristics and outcomes. Within-group changes in functional performance and prevalence of frailty risk factors between baseline and 1-year follow-up was examined using Wilcoxon signed-rank or paired sample *t*-tests and Mc Nemar's tests. Multiple logistic regression was performed to examine the independent effect of intervention and IC on prefrailty reversal, adjusted for age, gender and any significant univariate variables. Two separate models were compared—the first model included individual IC domains and the second model included composite IC score. Statistical analysis was performed using STATA SE 15.0 (Stata Corp., College Station, TX). All statistical tests were two-tailed, with *p-*value ≤ 0.05 considered statistically significant.

## Results

Among 209 participants with complete IC data and meeting operational criteria for prefrailty, only 81 were available for 1-year follow-up owing to restrictions imposed by the COVID-19 pandemic. Fifty two participants (64.2%) were enrolled in the multi-domain exercise and nutritional intervention programme, and 29 participants (35.8%) who declined intervention constituted the control group. There was no difference in age and composite IC score between participants who attended 1-year review compared with those who were excluded, although male participants were more likely to have been excluded albeit not statistically significant (21.0% vs. 32.8%, *p* = 0.064).

Median attendance at group-based exercise and nutritional sessions was 72.7% (interquartile range: 18.2–81.8). Weekly exercise target was achieved only for 5 (interquartile range: 1–9) weeks over the intervention period.

### Baseline characteristics

Intervention and control group participants were similar in age, gender, education level and comorbidity burden. Enrolment criteria based on FRAIL responses and measures of gait speed and grip strength were similar between groups. There was no difference in functional performance, life space mobility, and physical activity level at baseline between groups. Among measures representative of social vulnerability, participants in the intervention group were significantly more likely to report having insufficient expenses (38.5% vs. 17.2%, *p* = 0.047), and less likely to be in active employment (11.5% vs. 34.5%, *p* = 0.014). Impaired performance on the CMMSE was significantly more prevalent in the intervention group, contributing to significant decline in the cognition domain compared with control (17.3% vs. 0% with cognition domain score 0, *p* = 0.038). The intervention group also exhibited greater decline in the sensory domain (9.6% vs. 0% with sensory domain score 0, *p* = 0.032). Locomotion, psychological and vitality domains were similar between intervention and control groups, with both groups having similar composite IC scores (Table 1).

**Table 1:** Baseline characteristics.

	**Intervention (*N* = 52)**	**No intervention (*N* = 29)**	***p*-value**
**Demographics**			
Age	69.8 (6.2)	69.8 (7.0)	0.954
Gender (Female)	40 (76.9%)	24 (82.8%)	0.536
Education (≤6 years)	25 (49.0%)	17 (58.6%)	0.408
**Social**			
Lives alone	9 (17.3%)	5 (17.2%)	0.994
Lack confidant	8 (15.4%)	9 (31.0%)	0.097
No social contact	1 (7.7%)	0	0.291
No community activities	3 (10.3%)	5 (9.8%)	1.00
Inadequate expenses	20 (38.5%)	5 (17.2%)	0.047
Help others	13 (25.0%)	7 (24.1%)	0.931
Active employment	6 (11.5%)	10 (34.5%)	0.013
**Number of comorbidities**	2 (1–2.5)	1 (0–2)	0.101
Smoking (ex/current)	4 (7.7%)	5 (17.2%)	0.403
Alcohol	8 (15.4%)	3 (10.3%)	0.738
**Clinical characteristics**			
CMMSE impaired	9 (17.3%)	0	0.017
Depression	12 (23.1%)	5 (17.2%)	0.536
At-risk/malnourished	13 (25.0%)	5 (17.2%)	0.421
BMI (kg/m^2^)	24.7 (4.5)	23.2 (3.3)	0.117
Any fall past 1-year	15 (28.5%)	6 (20.7%)	0.422
**Functional performance**			
Barthel index	20 (20–20)	20 (20–20)	0.598
Lawton's IADL	23 (22–23)	23 (20–23)	0.863
Life space assessment	77.6 (25.0)	85.5 (17.7)	0.143
Physical activity (hours/week)	19.3 (17.6)	20.9 (18.0)	0.738
**Locomotion domain score**			0.758
0	13 (25.0%)	8 (27.6%)	
1	8 (15.4%)	6 (20.7%)	
2	31 (59.6%)	15 (51.7%)	
**Cognition domain score**			0.038
0	9 (17.3%)	0	
1	16 (30.8%)	14 (48.3%)	
2	27 (51.9%)	15 (51.7%)	
**Psychological domain score**			0.329
0	12 (23.1%)	5 (17.2%)	
1	4 (7.7%)	0	
2	36 (69.2%)	24 (82.8%)	
**Sensory domain score**			0.032
0	5 (9.6%)	0	
1	11 (21.2%)	13 (44.8%)	
2	36 (69.2%)	16 (55.2%)	
**Vitality domain score**			0.780
0	9 (17.3%)	4 (13.8%)	
1	11 (21.2%)	8 (27.6%)	
2	32 (61.5%)	17 (58.6%)	
**Composite IC**	7 (6–9)	8 (6–9)	0.719
Number domains impaired	2 (1–3)	2 (1–3)	0.600
**Enrolment criteria**			0.24
FRAIL –, weak and/or slow	23 (44.2%)	18 (62.1%)	
FRAIL +, unimpaired grip/ gait	12 (23.1%)	6 (20.7%)	
FRAIL +, weak and/or slow	17 (32.7%)	5 (17.2%)	
Slow gait speed	9 (17.3%)	3 (10.3%)	0.398
Weak grip strength	37 (71.2%)	23 (79.3%)	0.422

CMMSE, modified Chinese Mini Mental State Examination; IADL, instrumental activities of daily living.

### Outcomes

Sixteen (30.8%) participants in the intervention group no longer fulfilled operational criteria for prefrailty at 1-year, and reversal to robustness was also observed in 13 (44.8%) of control participants (*p* = 0.206). Individual frailty criteria by FRAIL items were similar between intervention and control groups, although the intervention group had significantly more participants with slow gait speed at follow-up. There was no difference between intervention and control groups in 1-year prevalence of cognitive impairment, depression and malnutrition (Table 2). However, within-group comparisons demonstrated significantly reduced prevalence of depression among participants in the intervention group at 1-year relative to baseline (7.8% vs. 23.1%, *p* = 0.022). Functional performance, life space mobility and physical activity level were similar between intervention and control groups at follow-up, and the measures were stable relative to baseline in both groups. While BMI was similar between groups at follow-up, control group participants registered a significant gain in BMI at 12-month relative to baseline (paired sample *t*-test *p* = 0.002). Both intervention and control groups exhibited significant improvement in grip strength at follow-up, with weak grip observed in 48.3% of intervention and 51.9% of control group participants at follow-up, relative to 71.2 and 79.3%, respectively at baseline (Mc Nemar's *p* = 0.004; *p* = 0.021). 1-year incidence of hospitalization was similar between groups, with a trend for lower falls incidence in the control group (*p* = 0.065).

**Table 2:** Frailty and clinical outcomes at 1-year.

	**Intervention (*N* = 52)**	**No intervention (*N* = 29)**	***p*-value**
Reversal to robustness	16 (30.8%)	13 (44.8%)	0.206
**Prefrailty characteristics**			0.603
FRAIL –, weak and/or slow	18 (34.6%)	7 (24.1%)	
FRAIL +, unimpaired grip/ gait	5 (9.6%)	2 (6.9%)	
FRAIL +, weak and/or slow	13 (25.0%)	7 (24.1%)	
Slow gait speed	7 (13.5%)	0	0.039
Weak grip strength	27 (51.9%)*	14 (48.3%)*	0.753
**FRAIL item responses**			
Fatigue_baseline_	5 (9.6%)	3 (10.3%)	0.916
Fatigue_12 − mth_	3 (5.8%)	1 (3.5%)	0.644
Resistance_baseline_	20 (38.5%)	6 (20.7%)	0.100
Resistance_12 − mth_	18 (34.6%)	7 (24.1%)	0.328
Ambulation_baseline_	7 (13.5%)	3 (10.3%)	0.683
Ambulation_12 − mth_	10 (19.2%)	2 (6.9%)	0.134
Loss of weight_baseline_	4 (8.0%)	4 (13.8%)	0.411
Loss of weight_12 − mth_	1 (1.9%)	1 (3.5%)	0.693
Illnesses_baseline_	0	1 (3.5%)	0.178
Illnesses_12 − mth_	0	0	–
**Clinical characteristics**			
CMMSE impaired	11 (21.6%)	4 (13.8%)	0.392
CMMSE score change	0.48 (2.22)	−0.28 (1.79)	0.120
Depression	4 (7.8%)*	3 (10.3%)	0.703
GDS score change	0.52 (1.91)	0.24 (0.31)	0.473
At-risk/malnourished	12 (23.1%)	7 (24.1%)	0.914
MNA-SF score change	0.006 (1.39)	0.03 (1.61)	0.946
BMI (kg/m^2^)	25.0 (4.8)	23.7 (3.5)*	0.231
BMI change	0.28 (1.24)	0.57 (0.91)	0.258
**Functional performance**			
Barthel index	20 (20–20)	20 (20–20)	0.408
Barthel index score change	0.13 (0.60)	0.17 (0.76)	0.805
Lawton's IADL	23 (22–23)	23 (22–23)	0.588
Lawton's score change	0.37 (2.31)	0.24 (2.23)	0.815
Life Space Assessment (LSA)	75.7 (21.8)	82.9 (16.1)	0.124
LSA score change	−1.82 (20.2)	−2.2 (21.4)	0.933
Physical activity (hours/week)	20.9 (15.2)	25.5 (19.9)	0.280
Physical activity change	1.66 (17.27)	6.37 (24.64)	0.375
**Health-related outcomes**			
Hospitalization	9 (17.3%)	4 (13.8%)	0.680
Falls	12 (23.1%)	2 (6.9%)	0.065

CMMSE, modified Chinese Mini Mental State Examination; IADL, instrumental activities of daily living.

^*^ McNemar's p < 0.05 for within group comparison between baseline and 12-month follow-up.

### Multiple logistic regression for prefrailty reversal, hospitalization and incident falls at 1-year

In Model 1 including age, gender, active employment status, baseline prefrailty enrolment criteria, and the cognition and sensory domains in isolation, receipt of multidomain exercise and nutrition intervention was not associated with prefrailty reversal. Older participants were significantly more likely to remain prefrail (OR = 1.14 95% CI 1.01–1.29, *p* = 0.034). Prefrail participants who were both FRAIL score positive and exhibited slow gait and/or weak grip strength at baseline were most likely to remain prefrail compared with those who were asymptomatic on FRAIL but enrolled due to slow gait speed and/or weak grip strength (OR = 6.89 95% CI 1.19–39.91, *p* = 0.031).

Neither cognition nor sensory domain was associated with prefrailty reversal.

Model 2 included age, gender, active employment status, baseline prefrailty enrolment criteria and composite IC score. Older age was associated with remaining prefrail although not statistically significant (OR = 1.12, 95% CI 1.00–1.27, *p* = 0.057). Higher composite IC at baseline was associated with reduced likelihood of remaining prefrail (OR = 0.67, 95% CI 0.45–1.00, *p* = 0.049). Again, intervention had no effect on prefrailty reversal (Table 3A).

In multiple logistic regression including age and gender, hospitalization was not associated with either intervention or composite IC. However, higher composite IC reduced risk of incident falls (OR = 0.72, 95% CI 0.51–1.00, *p* = 0.051), independent of intervention exposure (Table 3B).

**Table 3A:** Multiple logistic regression for prefrailty at 12-month.

	**Model 1**	**Model 2**
Intervention	2.23 (0.67–7.44), *p* = 0.191	1.90 (0.56–6.42), *p* = 0.304
Decline in cognition	0.47 (0.14–1.56), *p* = 0.551	
Decline in sensory	2.78 (0.72–10.68), *p* = 0.137	
Composite IC		0.67 (0.45–1.00), *p* = 0.049

Model 1 (R^2^ = 26.5%): adjusted for age, gender, employment status, baseline prefrailty enrolment criteria.

Model 2 (R^2^=27.2%): adjusted for age, gender, employment status, baseline prefrailty enrolment criteria.

**Table 3B:** Multiple logistic regression for incident falls.

	**Model 1**	**Model 2**
Intervention	3.86 (0.78–18.88), *p =* 0.097	3.59 (0.72–17.96), *p =* 0.120
Decline in cognition	1.44 (0.42–4.95), *p =* 0.562	
Decline in sensory	1.11 (0.40–3.06), *p =* 0.841	
Composite IC		0.72 (0.51–1.00), *p =* 0.051

Model 1 (R^2^ = 8.5%): adjusted for age, gender.

Model 2 (R^2^ = 13.4%): adjusted for age, gender.

## Discussion

In this first study to examine the role of intrinsic capacity in contributing to the outcomes of a prefrailty intervention programme, prefrailty reversal and falls incidence were independent of intervention exposure but influenced by baseline composite IC score. Reversal to a state of robustness, represented by a FRAIL score of 0 with unimpaired grip strength and gait speed, was observed in 30–40% of prefrail older adults from both intervention and control groups. Prefrailty reversal was driven by improvement in grip strength in both groups. The multi-domain exercise and nutritional intervention was associated with significant improvement in mood.

Natural transitions in frailty states have been well-explored, demonstrating the dynamic and bi-directional nature of the frailty syndrome (37). Our observed reversal rate of 30–45% over 1-year was higher than the reported spontaneous regression involving approximately 25% of prefrail older adults in recent meta-analyses with average follow-up of 3 to 4 years (38). However, prefrail participants of a combined exercise and nutritional intervention were not more likely to revert to robustness compared with their control group counterparts, contradicting available evidence supporting additive effects of multi-domain interventions in improving frailty characteristics and physical function, albeit in mixed prefrail and frail populations (12). Intervention trials for frailty commonly include both prefrail and frail older adults, but the delineation between prefrailty and frailty will be necessary as the extent of frailty may act as an effect modifier of interventions on frailty status (39). Our study adds to the limited literature focused specifically on prefrail older adults. With prefrailty defined using the Fried phenotype, Serra-Prat and colleagues reported that an intervention addressing nutrition and physical activity was effective in preventing frailty progression, but there was no significant difference in achieving reversal from being prefrail to robust at 1-year follow-up. Similar to our observations, there was no difference in individual frailty criteria between the intervention and control groups at follow-up (40). Although not subject to active exercise and nutritional intervention, participants in the control group had received individual counseling based on their screening results and group-based education focusing on exercise and nutrition for frailty prevention that was open to all participants post-screening. The potential effect of the counseling and education in the control group cannot be dismissed, considering the higher reversal rate compared with historical natural regression. Components of multi-domain interventions are highly variable across studies. In one multicomponent frailty prevention programme incorporating exercise, cognitive training and board game activities for prefrail older adults, the intervention group was significantly more likely to revert from being prefrail to robust by 12 weeks (41). Another multi-factorial, interdisciplinary intervention focused on physical exercise, dietary advice, review of polypharmacy and social assessment was significantly associated with reversal to robustness in prefrail elderly (42). The general consensus for prefrailty as an intermediate multidimensional risk state associated with physical impairment, cognitive deficits, malnutrition and social vulnerability (15) further support the utility of complex and targeted management strategies beyond a conventional exercise and nutrition approach to optimize its reversal.

Decline in IC was highly prevalent in our cohort of prefrail older adults, affecting 90.1% of the cohort at baseline. Both intervention and control group participants averaged losses in 2 domains. This observation reinforces earlier findings suggesting the significant public health problem of IC impairment (5, 43). The present study builds on the emerging evidence for the relationship between IC and frailty, as composite IC rather than intervention exposure dictated reversal from being prefrail to robust. Interestingly, composite IC score, but not the individual domains, was predictive of prefrailty reversal. This may be attributed to the integrative nature of the IC construct, such that a global score may better reflect the physical and mental capacities, and being more informative in identifying at-risk older adults for tailored preventative care. A recent study suggested that IC trajectories were more likely to parallel frailty transitions among robust and prefrail older adults, while significant losses that have already culminated in a frail individual render it more challenging to seek IC improvement for frailty reversal (44). With declining IC anticipated with age, the monitoring of IC among non-frail older adults can provide opportunities for intervention to reverse the trend and prevent or delay frailty onset.

The multidomain exercise and nutritional intervention had a positive impact on mood, evident by the 15% reduction in prevalence of depression at 1-year among participants in the intervention group. Our results are consistent with reported benefits of physical activity on depressive symptomatology in older adults (45). With social support being a strong predictor of depressed mood among community-dwelling older adults (46), the contacts established through the group-based intervention may have contributed to improvement in mental health. Beyond the psychosocial effects, exercise may influence mood through biological mechanisms including increased neurotrophic factors in circulation, anti-inflammatory effects, reduced oxidative stress and neuroendocrine regulation (47). Despite the focus on nutrition, nutritional status remained unchanged, with approximately one-quarter of participants in intervention and control groups, respectively, being assessed to be at-risk of malnutrition or malnourished at follow-up. Our nutritional intervention emphasized dietary habit change without provision of supplementation, which may be inadequate in the setting of malnutrition. This is consistent with the findings from the Prefrail 80 study, in which a group session on the Mediterranean diet as part of a multifactorial intervention failed to impact on nutritional status, and worsening nutritional status over time was observed among those who progressed to frailty (41). It should be cautioned that the findings do not imply routine use of oral nutritional supplements, which should be considered only for frail older adults presenting with weight loss or malnutrition (48). Thus, the assessment of IC may better guide a targeted approach toward prefrailty/frailty, based on the identified domain losses. For example, ICOPE recommends oral nutritional supplementation with increased protein intake for older adults who are malnourished (4). The reliance on BMI for monitoring intervention should also be cautioned as we observed significant gain in BMI among control group participants even as nutritional status remained unchanged.

Contrary to the extant literature supporting exercise for falls prevention in older adults, we observed a non-significant trend for increased falls risk in the intervention group. There was no change to physical activity level or life space to account for the increased exposure to risk. However, participants in the intervention group were more likely to exhibit significant declines in the cognition and sensory domains, both of which constitute intrinsic risk factors for falls (49). This was supported in the multiple logistic regression, whereby composite IC rather than intervention predicted fall risk during follow-up.

We acknowledge several limitations. Among studies restricted to a prefrail cohort, rates of prefrailty reversal were variable, influenced by follow-up duration, intervention intensity and definition of prefrailty (40, 41). Even with conservative estimates from these studies, an overall sample of at least 164 prefrail participants would be needed assuming a difference of 50% in prefrailty reversal between groups using two-sided sample size calculation with type II error of 0.80. Thus, our sample size of 81 prefrail older adults was statistically underpowered for the reversal of prefrailty status as a primary outcome. This was a pragmatic trial with a convenient control group of participants who declined intervention. The reasons for declining intervention were not tracked, although we noted that participants who declined and constituted the control group were more likely to be actively employed, and social and physical activities associated with employment status might not have been comprehensively captured by the social questionnaire and PAVS. However, even after adjusting for employment status, intervention exposure was not associated with prefrailty reversal. The suboptimal adherence at 70% to the overall programme could have contributed to the lack of intervention effect. Further, compliance with individually prescribed home exercises was generally poor, with an average of 6 weeks in the 4-month intervention period fulfilling the weekly exercise target of 3 days per week. The observed compliance was consistent with a recent review of interventions for frailty, with adherence varying between 47.5 and 90.4%, and poorer for home-based interventions (50). Additionally, sustainability of exercise and nutritional habits of the intervention group participants beyond the 4-month intervention period was not monitored. The COVID-19 pandemic yielded substantial disruption, such that only 40% were available for 1-year follow-up, although age and composite IC were similar between cohorts included and excluded from this analysis. While validated and objective measures for the assessment of IC were employed for the locomotion, cognition, psychological and vitality domains, both hearing and vision relied on subjective reports.

In conclusion, IC decline is highly prevalent among prefrail older adults. The multidomain exercise and nutritional intervention focuses on the locomotion and vitality domains, and may not adequately address component domain losses to optimize prefrailty reversal. Future studies should consider investigating the effect of multidomain interventions on global IC measure, and the impact that addressing IC may have on frailty transitions.

## Data availability statement

The original contributions presented in the study are included in the article/supplementary material, further inquiries can be directed to the corresponding author/s.

## Ethics statement

The studies involving human participants were reviewed and approved by SingHealth Institutional Review Board. The patients/participants provided their written informed consent to participate in this study.

## Author contributions

LT contributed to overall study design, conducting the study, and analysis and main manuscript writing. E-LT, SM, and AL contributed to intervention design, data collection, and conducting the study. Y-SN contributed to study design and manuscript writing. All authors reviewed the final manuscript. All authors contributed to the article and approved the submitted version.

## Funding

This study was funded by National Medical Research Council Centre Grants (CGAug16C027 and CGAug16M0) and National Innovation Challenge on Active and Confident Ageing (MOH/NIC/HAIG04/2017). The grants funded the research staff, assessment equipment and on-site conduct of the trial, and the researchers were independent from funders.

## Conflict of interest

The authors declare that the research was conducted in the absence of any commercial or financial relationships that could be construed as a potential conflict of interest.

## Publisher's note

All claims expressed in this article are solely those of the authors and do not necessarily represent those of their affiliated organizations, or those of the publisher, the editors and the reviewers. Any product that may be evaluated in this article, or claim that may be made by its manufacturer, is not guaranteed or endorsed by the publisher.
